# Bifocal malakoplakia in a patient living with HIV: case report

**DOI:** 10.1186/s12981-024-00592-w

**Published:** 2024-01-07

**Authors:** Mohammed Alsaeed, Mohamed Mursi, Nazik Eltayeb, Hadi Kuriry, Salafa Albaghli, Yasir Alrusayni

**Affiliations:** 1 Medicine department, Infectious disease division, Prince Sultan Military Medical City, P.O.Box 7897, Riyadh, 11159 Saudi Arabia; 2 Multi Organ Transplant Center of Excellence, King Fahad Specialist Hospital, Dammam, Saudi Arabia; 3 Pathology department, Prince Sultan Military Medical City, Riyadh, Saudi Arabia; 4 Collage of Medicine, Alfaisal University, Riyadh, Saudi Arabia

**Keywords:** Malakoplakia, HIV, CMV, Rhodococcus equi, SARS-CoV-2

## Abstract

**Background:**

Malakoplakia is a rare chronic granulomatous disease characterized by the presence of Michaelis-Gutmann bodies (MGBs) within histiocytic aggregates. It predominantly affects immunocompromised individuals, including those living with Human Immunodeficiency Virus (HIV).

**Case Presentation:**

We present a unique case of bifocal malakoplakia in a 49-year-old man, previously with Coronavirus disease 2019 (COVID-19) and HIV positive, presented with respiratory symptoms, weight loss, and lymphadenopathy. He had various infections including Non-Tuberculous Mycobacteria (NTM), Cytomegalovirus (CMV), and Candida, with evolving lung and gastrointestinal issues. Despite treatment attempts, he deteriorated due to respiratory distress, multi-organ failure, and coagulopathy, leading to his unfortunate demise.

**Conclusion:**

This report presents a distinctive and complex case of malakoplakia in an HIV-positive patient, a rare inflammatory disorder originally described by Michaelis and Gutmann in 1902. The hallmark Michaelis-Gutmann organisms were observed, confirming the diagnosis. While typically affecting the urinary tract, this case demonstrates the exceptional ability of malakoplakia to manifest in various organ systems, including pulmonary, gastrointestinal, and more. Although Escherichia coli is a prevalent associated pathogen, the exact cause remains elusive. Treatment, often involving surgical excision and antibiotic therapy, underscores the challenging nature of managing this condition in immunocompromised individuals.

## Background

Malakoplakia, an infrequent inflammatory disorder, poses diagnostic and therapeutic challenges, particularly in immunocompromised individuals. First identified in 1902, it is characterized by the presence of Michaelis-Gutmann bodies, pathognomonic for the condition [1]. While commonly observed in the urinary tract, malakoplakia’s ability to affect diverse organ systems, such as the pulmonary, gastrointestinal, and nervous systems, underscores its enigmatic nature [2, 3]. Escherichia coli is often implicated, though its etiology remains unclear [4].

Treatment strategies frequently involve surgical intervention and antibiotics, reflecting the complex management required. This report highlights a unique case of malakoplakia in an HIV-positive patient, emphasizing its potential to manifest beyond its usual parameters and the intricate task of addressing this condition in immunocompromised settings.

## Case presentation

**Fig. 1:**
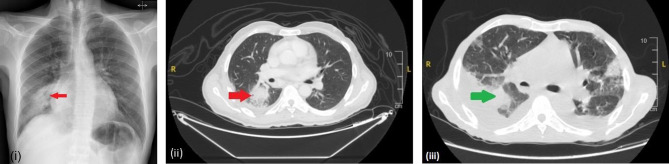
Image (**i**) A chest radiograph upon arrival of the first admission, demonstrates a right lower zone infiltration with a small cavitary lesion. Image (**ii**) CT chest of the first admission, demonstrate a 2.7 × 3 cm heterogenous enhancing soft tissue lesion compressing the right lower lobe segmental bronchus (red arrows). Image (**iii**) CT Chest of the second admission, show the evolving right lower lobe mass reaching 10.3 × 10.5 cm making more compression on the right lower segmental main bronchus (green arrows) with the presence of air pockets as demonstrated as shown in images

A 49-year-old man reported to the emergency room (ER) on March 16, 2021, with one month of shortness of breath, productive cough, and fever, as well as a nine-kilogram weight loss. He had mild COVID19 pneumonia three months before admission. His temperature was 38.1 ^o^C, blood pressure 101/52, pulse rate 108 beats per minute, respiratory rate 20 breaths per minute, and ambient air oxygen saturation 99%. He looked malnourished and pail, he had finger clubbing. The right lung had fine crepitations, and the submandibular and cervical lymph nodes were palpable but not painful. Table (1) shows his initial lab tests. A chest radiograph showed a right sided homogenous infiltration with a cavitary lesion at the same site as shown in Fig. (1).The patient was admitted with pneumonia and has been given piperacillin-tazobactam 4.5gm every 6 h and azithromycin 500 mg once daily empirically. SARS-CoV-2 PCR was positive; sputum culture revealed susceptible *pseudomonas aeruginosa*; ceftazidime 2gm every 8 h replaced piperacillin-tazobactam.

**Table 1:** Laboratory results

Test/ Normal range	First admission March 2021	Second Admission June 2021
WBCs (4-1110^9/l)	10.1	9.04
Hemoglobin (125–180)	86 g/L	54 g/L
Mean cell volume(75–95)	85 fl.	88 fl.
Platelets count (150–350 1110^9/l)	342	143
Neutrophils count (1.8–7.5 10^9/l)	8.8	7.8
Lymphocytes count (1.5-4.0 10^9/l)	0.6	0.39
Sodium (136–145)	133 mmol/L	131 mmol/L
Potassium (3.5–5.1)	3.5 mmol/L	3.4 mmol/L
Urea (2.8–8.1)	6.6 mmol/L	6.0 mmol/L
Creatinine(59–104)	51 mmol/L	51 mmol/L
Corrected Calcium (2.15–2.5)	2.62 mmol/L	2.42 mmol/L
Albumin (35–52)	21 g/L	17 g/L
Total bilirubin (2–21)	20 umol/L	11 umol/L
Alkaline phosphatase(40–129)	511 U/L	447 U/L
Alanine aminotransferase(5–41)	60 U/L	23 U/L
Gamma GT (8–61)	189 U/L	Not done
C-reactive protein (0–6)	160 mg/L	117.36 mg/L
ESR (0–15)	113 mm/hr	102 mm/hr
Procalcitonin (≤ 0.5)	0.48 ug/L	0.34 ug/L
COVID19 PCR	Positive	Positive
ANA	Positive	Not done
ANCA	Positive p-ANCA Negative c-ANCA	Not done

As a result of elevated liver function tests, abdominal ultrasonography was performed, which revealed diffuse hepatic steatosis. Human immunodeficiency virus (HIV) antibody test was done that turned to be positive.In addition to primary sclerosing cholangitis caused by Epstein-Barr virus (EBV) versus cytomegalovirus (CMV), mycobacterial infections were suspected. The patient’s CD4 count was 20 cells/cu mm, while the CMV and EBV viral loads were 659,072 and 81 IU/mL, respectively. HIV viral load and genotypic analysis were unavailable at the time. Negative results have been reported for the syphilis, hepatitis C virus serology, and hepatitis B surface antigen. Magnetic resonance cholangiopancreatography was done that showed no intra or extra hepatic biliary obstruction in addition to a 0.5 × 0.5 cm lesion in segment 7 in keeping with haemangioma.

As verified by the ophthalmology team, the patient lacked clinical evidence of CMV retinitis, and he was started on valganciclovir 900 mg twice daily in addition to Co-trimoxazole 1 double strength three times per week as prophylaxis. As shown in Fig. (1), a computed tomography (CT) off the chest revealed a 2 by 3 cm mass in the right lower lobe at the right main bronchus with ground glass opacity and collapse. The patients underwent bronchoalveolar lavage (BAL) without a biopsy. The procedure revealed a 2 by 3 cm polyp in the right bronchus. In addition to cytology and mycobacterium tests, BAL samples were sent for bacterial and fungal culture. The results are listed in Table (2). The patient went home on valganciclovir, Co-trimoxazole as prophylaxis and ciprofloxacin 750 mg twice daily until review his pending laboratory results at the virology outpatient clinic.

**Table 2:** Bronchoalveolar lavage (BAL) results of the first admission

Bacterial culture	No growth
Fungal culture	Candida glabrata
Acid fast bacilli smear	Three sample were taken and two turned to be weakly positive
TB PCR	Negative
TB culture	Pending (turned to be positive for Non-Tuberculous Mycobacterium after 8 weeks in August 2021 and the patient passed away in July 2021).
Respiratory panel PCR	Negative
Cytology	Negative for malignancy and special fungal stain turned to be negative.

On the basis of clinical presentation and two positive acid-fast bacilli samples with negative TB PCR, the patient was diagnosed with nontuberculous mycobacterium infection at the virology clinic on 11 April 2021. As a result, ethambutol 1.2 g once daily, clarithromycin 500 mg twice daily, and rifampicin 600 mg once daily were added to valganciclovir 900 mg twice daily, Co-trimoxazole 960 mg three times weekly, and ciprofloxacin 750 mg twice daily. The patient was given a three-week follow-up appointment and advised to repeat sputum for acid-fast bacilli, liver function test, computed tomography of the chest, and CMV viral load. The patient missed his appointment and showed up on May 30, 2021. On that visit, his symptoms did not improve; however, he had trouble being adherent with his medication. While ciprofloxacin was discontinued, he was instructed to continue taking valganciclovir, Co-trimoxazole, ethambutol, clarithromycin, and rifampicin.On June 13, 2021, he returned to the clinic with progressive weight loss and cough as he struggled to adhere to his medication. Genvoya (Elvitegravir 150 mg, cobicistat 150 mg, emtricitabine 200 mg, tenofovir alafenamide 10 mg) was prescribed, and the patient was instructed to adhere to his medication. HIV viral load, CMV viral load, lymphocyte analysis, and computed tomography of the chest and abdomen were requested.

On June 21, 2021,the patient presented to the ER with complaints of progressive fatigue, poor appetite, fever, generalized body pain, and congestion, as well as poor medication adherence.He reported that he wasn’t taking Genvoya and was not compliant with all medications. Examination showed a severely cachectic man with severe pallor. The preliminary investigations are outlined in Table (1). Meropenem 1 gm every 8 h and ganciclovir 250 mg twice daily were added to Co-trimoxazole prophylaxis, while other medications were discontinued. Despite inadequate medication adherence, the CMV viral load decreased to 1,592 IU/mL from 659,072 IU/mL in March 2021, and the HIV viral load was 3,541 IU/mL.

**Fig. 2:**
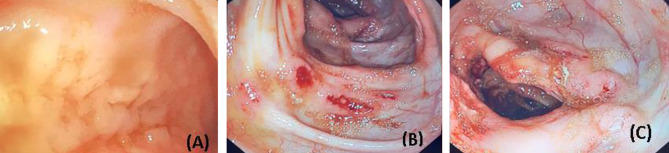
Gross endoscopic appearance of the right colon showing the cecal base at ileocecal valve with deformed looking. Multiple ulcers ranging between 5 mm and 1 cm in diameter with noticed with raised edges and whitish base. Some ulcers were oozing blood and others were covered by clots

Due to severe anaemia, packed red blood cells were given. The patient underwent upper and lower gastrointestinal (GI) endoscopies. The upper GI scope revealed only mild gastritis, whereas the lower GI scope revealed extensive ulcers, nodules, and erythematous changes involving the right colon. As a result, several samples were taken from the right and left sides of the colon for full analysis. In Fig. (2), images of lower GI endoscopy are showed.

**Fig. 3:**
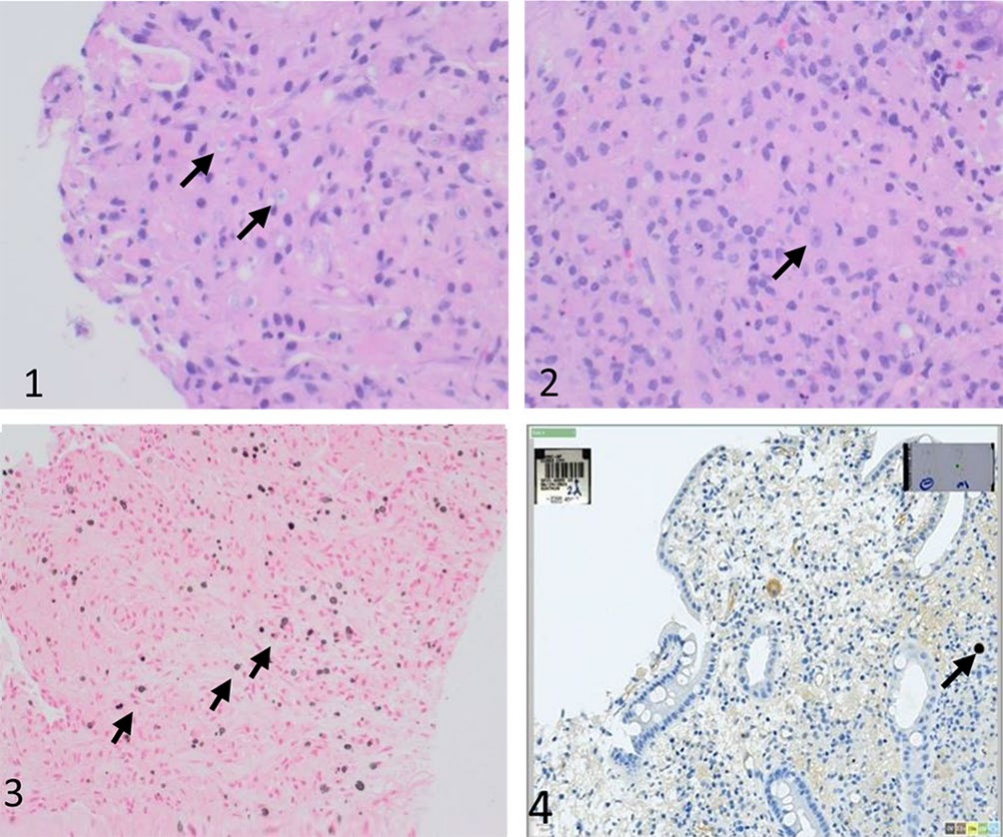
(**1**) Malakoplakia. High magnification view showing epithelioid histiocytes with abundant eosinophilic cytoplasm and intracytoplasmic round basophilic targetoid inclusions (Michaelis-Gutmann bodies) (arrows), diagnostic of malakoplakia(Lung). (**2**) Malakoplakia. High magnification view showing colonic epithelium with underlying epithelioid histiocytes (arrow) with abundant eosinophilic cytoplasm(Colon). (**3**) The Michaelis-Gutmann bodies are highlighted with a von Kossa stain. (**4**) Immunostaining for CMV is POSITIVE

CT of the chest and abdomen revealed a progression of right lower lobe mass and worsening lung parenchymal disease with bilateral effusion and a small pericardial effusion, as depicted in Fig. (1). The expanding right lung mass was biopsied using ultrasound guidance, and samples were sent for microbiology and histology evaluation. TB culture from the lung mass was pending at the time, while PCR and three acid-fast bacilli smears came back negative. *Candida krusei* was isolated from the lung mass, so voriconazole treatment was initiated. As shown in Fig. (3), the right colon histological sample demonstrated malakoplakia with positive CMV stains while lung showed malakoplakia only.

Ascorbic acid was added to the patient’s medication. He improved clinically and requested discharge. Before discharge, ganciclovir was replaced with valganciclovir, co-trimoxazole continued three times per week, and voriconazole continued 200 mg twice day with ascorbic acid. Descovy (emtricitabine and tenofovir) and Dolutegravir were also given. He tolerated his medicines well and discharged home with two weeks follow up in virology clinic.

10 days later, patient returned to ER with worsening shortness of breath, poor oral intake, and acute renal injury. He was unstable and started double inotropic support. SARS-CoV-2 was isolated again. The right lung biopsy sample from the previous admission showed malakoplakia with *Rhodococcus equi* positive culture. The patient received broad-spectrum antimicrobials. After three days, the patient passed away he developed acute respiratory distress syndrome, multi-organ failure, and severe coagulopathy.

## Discussion

We report the first case of bifocal malakoplakia in an HIV-positive individual. Malakoplakia is a rare granulomatous disease that is characterized by the accumulation of Michaelis-Gutmann bodies (MG) in affected tissues. MG bodies are large, round, basophilic inclusions that are composed of degenerating leukocytes, bacteria, and cellular debris [1]. Malakoplakia most commonly affects the urinary tract, but it can also involve other organs, such as the liver, lungs, skin, and bones. Although malakoplakia can occur in immunocompetent individuals, it is more commonly associated with immunosuppressive conditions, such as HIV infection [2].

Although the exact cause of malakoplakia is unknown, it is believed that macrophages’ capacity to kill and digest bacteria is impaired. The lack of the enzyme lysosome-associated membrane protein-3 (LAMP-3) is thought to be the cause of this poor ability to kill bacteria. LAMP-3 is essential for the fusion of lysosomes with phagosomes, which are the structures that contain ingested bacteria. When LAMP-3 is deficient, phagosomes are not properly digested, and the bacteria are able to survive and multiply. In patients with HIV, the compromised immune system leads to the dysfunction of macrophages, impairing their ability to eliminate bacteria effectively [3].

The most common organism associated with malakoplakia is *Escherichia coli*, but other bacteria, such as *Klebsiella pneumoniae* and *Proteus mirabilis*, have also been implicated [4]. Based on our review of the literature, we identified twenty-five HIV patients with proven malakoplakia Table 3 [5–23]. All had acquired immunodeficiency status. The condition primarily affects young to middle-aged individuals, with a 23:2 male-to-female ratio. As was the case with our patient, the lung was the most affected organ, and Rhodococcus equi was the most common pathogen. A unique feature of our patient is that his colon sample had CMV evidence in addition to malakoplakia, which we couldn’t find in similar cases.

**Table 3:** Proven HIV cases with malakoplakia

Reference	Age	Sex	Country	Immune status/ CD4 count cells/ cubic millimeter	Symptoms	Location	Culture	Medication received	Outcome
[5]	52	Male	Czech Republic	AIDS/ not reported	Productive cough and fever	Pulmonary	Rhodococcus equi	Not reported	Not reported
[6]	41	Male	United States	AIDS/ 44	Mass on scalp	Brain	No organism identified	Ciprofloxacin, Trimethoprim-sulfamethoxazole	Cure
[7]	39	Male	France	AIDS/ 5	Fever, weight loss, chronic diarrhea	Colon	Shigella boydii	Clarithromycin, ciprofloxacin	Cure
[8]	25	Male	Mexico	AIDS/ 7	Productive cough, weight loss	Pulmonary	Rhodococcus equi	Rifampicin, vancomycin, ciprofloxacin	Cure
[9]	45	Female	India	AIDS/ 173	Skin ulcer	Cutaneous	Staphylococcus Aureus	Ciprofloxacin	Cure
[10]	30	Male	United States	AIDS/ 35	Productive cough, weight loss and fever	Pulmonary	Rhodococcus equi	Rifabutin, azithromycin	Lost follow up
[11]	47	Male	United States	AIDS/ 33	Stridor and non- productive cough	Trachea	Rhodococcus equi	Rifampicin, azithromycin	Relapsed
[12]	45	Male	United States	AIDS/ not reported	Not available	Pulmonary	Rhodococcus equi	Not reported	Cure
[13]	25	Male	United States	AIDS/ not reported	Dyspnea, fever, cough, weight loss	Pulmonary	Rhodococcus equi	Rifampicin, erythromycin	Cure
[14]	49	Male	France	AIDS/ not reported	Not available	Pulmonary	Rhodococcus equi	Erythromycin, netilmicin	Died
[15]	45	Male	Canada	AIDS/ not reported	Not avialble	Pulmonary	Rhodococcus equi	Vancomycin, imipenem, doxycycline, erythromycin	Cure
[16]	40	Male	South Korea	AIDS/ not reported	Not avialble	Pulmonary	Rhodococcus equi	Not reported	Not reported
	45	Male	South Korea	AIDS/ not reported	Not avialble	Pulmonary	Rhodococcus equi	Not reported	Not reported
	50	Male	South Korea	AIDS/ not reported	Not avialble	Pulmonary	Rhodococcus equi	Not reported	Not reported
	50	Male	South Korea	AIDS/ not reported	Not avialble	Pulmonary	Rhodococcus equi	Not reported	Not reported
[17]	34	Male	France	AIDS/ not reported	Not avialble	Pulmonary	Rhodococcus equi	Vancomycin, imipenem, rifampicin, clarithromycin, teicoplanin	Cure
[18]	36	Female	United States	AIDS/ not reported	Cough, Fever, dysphagia	Pulmonary	Rhodococcus equi	Vancomycin, erythromycin	Lost follow up
[19]	49	Male	United States	AIDS/ not reported	Cough, fever, fatigue	Pulmonary	Rhodococcus equi	Ciprofloxacin	Lost follow up
[20]	37	Male	United States	AIDS/ not reported	Cough, fever, fatigue	Pulmonary	Rhodococcus equi	Not reported	Died
	48	Male	United States	AIDS/ not reported	Cough, fever, chest pain	Pulmonary	Rhodococcus equi	Not reported	Died
[21]	33	Male	United States	AIDS/ not reported	Not avialble	Pulmonary	Rhodococcus equi	Ciprofloxacin, erythromycin	Died
	41	Male	United States	AIDS/ not reported	Not avialble	Pulmonary	Rhodococcus equi	Vancomycin, erythromycin	Died
	43	Male	United States	AIDS/ not reported	Not avialble	Pulmonary	Rhodococcus equi	Erythromycin	Died
[22]	23	Male	Brazil	AIDS/ not reported	Not avialble	Pulmonary	Rhodococcus equi	Erythromycin	Lost follow up
[23]	29	Male	Spain	AIDS/ not reported	Fever, hemoptysis	Pulmonary	Rhodococcus equi	Imipenem, rifampicin, ciprofloxacin, doxycycline	Cure

Histopathological evaluation is essential for diagnosing malakoplakia. When you look at macrophages through a microscope, you can see Michaelis-Gutmann bodies, which are laminated structures stained with calcium. These bodies consist of concentric rings composed of calcium and iron, surrounded by basophilic fibrillar material. Immunohistochemical staining for CD68, and lysozyme can also aid in confirming the diagnosis [24].

Optimal management of malakoplakia in patients with HIV remains poorly understood due to the limited number of reported cases. Early recognition and the initiation of targeted therapy are essential to achieving favorable outcomes. In our case, the patient’s poor medication adherence and disease progression posed significant challenges in managing the complex infectious processes. Multidisciplinary collaboration among infectious disease specialists, histopathologists, gastroenterologists, and pulmonologists played a crucial role in the diagnosis and management of this rare condition.

The prognosis for malakoplakia is generally good with appropriate treatment. However, the disease can be recurrent, and patients with underlying medical conditions may have a worse prognosis.

## Conclusion

In conclusion, we present a rare case of bifocal malakoplakia in a patient living with HIV. Prompt recognition, multidisciplinary collaboration, optimal antimicrobial therapy, and adherence to treatment regimens are vital to achieving favorable outcomes in these challenging cases. Further research is warranted to better understand the pathogenesis, optimal management strategies, and prognosis of malakoplakia in patients with HIV.

## Data Availability

The data that support the findings of this study are openly available.
